# Echocardiographic Changes in Saudi Patients with Type 2 Diabetes Mellitus

**DOI:** 10.3390/medicina59111985

**Published:** 2023-11-10

**Authors:** Turky H. Almigbal, Dina S. Almunif, Khaled H. Aburisheh, Mazen M. Barhoush, Reem A. Aldhahi, Mohammed J. Anabi, Obeed A. Alotaibi

**Affiliations:** 1 Department of Family and Community Medicine, College of Medicine, King Saud University, Riyadh P.O. Box 11495, Saudi Arabia; d.almunif@gmail.com (D.S.A.);; 2 University Diabetes Centre, King Saud University Medical City, King Saud University, Riyadh P.O. Box 11495, Saudi Arabia; kaburisheh@ksu.edu.sa (K.H.A.);; 3 King Abdulaziz University Hospital, King Saud University Medical City, King Saud University, Riyadh P.O. Box 11495, Saudi Arabia

**Keywords:** diabetes mellitus, diabetes cardiomyopathy, echocardiography

## Abstract

*Background and Objectives*: Cardiovascular disease is one of the leading causes of morbidity and mortality among the diabetic population. Given the high prevalence of diabetes mellitus (DM) in Saudi Arabia and the high prevalence of heart failure in the diabetic population, this study assesses the echocardiographic changes in Saudi patients with type 2 DM (T2DM) compared with healthy controls. *Materials and Methods*: In this retrospective case–control study, 80 patients with diabetes (45 males, age: 58.78 ± 10.2 years) were compared with 80 controls (45 males, age: 58.6 ± 10 years) who underwent an echocardiographic study in the King Saud University Medical City, Riyadh, Saudi Arabia. *Results*: There were no significant differences between the patients with diabetes and controls in terms of aortic root diameter, left atrium diameter, posterior wall, interventricular wall thickness, left ventricular diameters and ejection fraction. However, diastolic dysfunction was statistically significantly higher in the diabetic group than in the control group (*p* < 0.05). *Conclusions*: This is the first case–control study in Saudi Arabia that assesses echocardiographic parameters in T2DM patients. DM is an independent risk factor for diastolic dysfunction regardless of its association with hypertension and dyslipidemia.

## 1. Introduction

Diabetes mellitus (DM) is one of the most common chronic metabolic diseases, and, in recent decades, DM rates have significantly increased worldwide. The Saudi population has the second highest prevalence of diabetes in the Middle East and is ranked seventh in the world for diabetes rates reported by the World Health Organization, with a prevalence of 25.4% [1]. It has been estimated that 40.3% are unaware of their disease [1] and are thus prone to developing diabetes complications. DM complications significantly increase morbidity and mortality. Cardiovascular disease (CVD) is the leading cause of death among the diabetes population [2,3,4].

Echocardiography plays an important role in the diagnosis of cardiomyopathy. It is the gold standard for providing a comprehensive evaluation of myocardial structure and function [5,6], and it is a valuable tool for facilitating early diagnosis and, hence, early medical management. It is widely used due to its proven safety and non-invasiveness.

It is well established that the heart is affected by DM. DM can contribute to CVD development through multiple factors, such as accelerated atherosclerosis, increased oxidative stress, endothelial dysfunction and cardiac autonomic neuropathy [7]. However, it has also been introduced as a contributory risk factor to heart failure, a different entity of heart disease. This is supported by the reported prevalence of HF in patients with diabetes being significantly higher than that reported in the general population. The prevalence in the diabetic population is 12%, whereas, in the general population, it is 1% to 4% [8]. Researchers of the Framingham study reported that diabetic women and men have a two- and five-fold increased risk, respectively, of congestive heart failure compared with the non-diabetic population [9]. However, it is unclear whether diabetes is a direct causative risk factor or related to confounders, such as coexistent hypertension, ischemic or valvular heart disease.

Controversy has emerged concerning the effect of DM on echocardiographic parameters [10,11]. To the best of our knowledge, the relationship between DM and cardiac structural changes has not been studied in Saudi patients with type 2 DM (T2DM) without overt heart disease. Given the high prevalence of DM in Saudi Arabia, we examine the direct effect of DM on the heart and detect any myocardial changes via an echocardiographic study of a Saudi diabetic population and a healthy population.

## 2. Materials and Methods

### 2.1. Study Design and Setting

This is a retrospective case–control study of 80 T2DM patients in King Saud University Medical City’s diabetes clinics and 80 control subjects (age- and gender-matched) who were identified as non-diabetic in the same hospital; both groups underwent an echocardiographic study. Approval for this study was obtained from the Institutional Review Board, College of Medicine, King Saud University (project no.: E-21-5899), Riyadh, Saudi Arabia. The patients were selected using the systematic random sampling technique; their data were retrieved from electronic medical records (EMRs) using a structured data clinical sheet, which included demographic data, clinical history and vital signs (weight, height, body mass index, systolic blood pressure (SBP) and diastolic blood pressure), as well as metabolic laboratory parameters, including total cholesterol (TC); low-density lipoprotein cholesterol (LDL-C); high-density lipoprotein cholesterol (HDL-C); triglyceride; creatinine; hemoglobin A1c (HgA1c); fasting blood glucose (FBG); and estimated glomerular filtration rate (eGFR), which was calculated using the CKD-EPI equation.

### 2.2. Data Collection Process

The data for this study were obtained through a systematic process of retrieving information from EMRs using a structured data clinical sheet, specifically designed to capture a comprehensive set of variables pertinent to the research objectives. The following steps outline the data collection process: defining data elements, creating the structured data clinical sheet, accessing EMRs, data extraction, quality assurance, data storage and management, and data validation.

### 2.3. Inclusion Criteria

This study included adult men and women aged between 18 and 75 with T2DM diagnosed based on ADA guidelines who underwent an echocardiographic study from 2018 to 2020, and they were compared with a healthy control group.

### 2.4. Exclusion Criteria

This study excluded patients aged 75 years or older and 18 years or younger; patients with type 1 DM or gestational diabetes mellitus; and patients previously diagnosed with comorbidities, such as heart failure, coronary artery disease, valvular heart disease or chronic kidney disease (eGFR of <30 mL/min/1.73 m^2^). This was documented in the patient history in the medical records.

### 2.5. Echocardiographic Evaluation

Transthoracic echocardiographic imaging was performed using a PHILIPS EPIQ 7 Ultrasound system (Philips, Amsterdam, The Netherlands), and the following parameters were obtained for each subject:

1. Aortic root diameter (ARD; 20–29 mm);
2. Left atrium diameter (LAD; 15–37 mm);
3. Left ventricular internal diameter end diastole (LVIDd; 35–56 mm);
4. Left ventricular internal diameter end systole (LVIDs; 22–40 mm);
5. Posterior wall thickness (PWT; 7–12 mm);
6. Interventricular wall thickness (IVWT; 7–11 mm);
7. Ejection fraction (EF; >50%).

A left ventricular diastolic dysfunction (LVDD) diagnosis was made by specialists in echocardiography based on the diagnostic criteria of the American Society of Echocardiography and the European Association of Cardiovascular Imaging (annular e0 velocity: septal e0 < 7 cm/s and lateral e0 < 10 cm/s; average E/e0 ratio > 14, LA volume index > 34 mL/m^2^ and peak TR velocity > 2.8 m/s).

### 2.6. Statistical Analysis

An analysis was performed using IBM-SPSS version 25. Descriptive statistics are presented as frequency, percentage, mean and standard deviations. For inferential statistics, chi-square was used to study the association between two categorical variables. A proportionality test and independent *t*-test were used to compare the diabetic and non-diabetic groups by percentage or mean. Categorical variables with a chi-square *p*-value less than 0.05 were further analyzed using multivariate logistic regression to determine the risk factor or the odds ratio of each factor. A statistical test *p*-value of less than 0.05 was considered significant.

### 2.7. Sample Size

The sample size for our study was 80 case patients with 80 matched controls, which was calculated using the epiR sample size for matched case–control studies. The calculation was conducted based on matched sets of cases and controls with 1 matched control(s) per case, and the probability of exposure among controls was 50%, with a correlation coefficient for exposure between matched cases and controls of 0.2. The odds ratio for disease in exposed subjects relative to unexposed subjects was 3, assuming that the Type I error probability was 5% [12].

## 3. Results

### 3.1. Characteristics of the Subjects

A total of 80 patients with diabetes (cases) and healthy subjects (controls) who underwent an echocardiographic study were enrolled in this study. Patients with T2DM had a mean age of 58.78 ± 10.2 years, the controls had a mean age of 58.6 ± 10 years, and both had the same gender proportions: 45 males (56.3%) and 35 females (43.8%). The mean duration of DM among patients was 13 ± 8.3 years. Compared to controls, patients with T2DM had a higher prevalence of hypertension (HTN) and dyslipidemia (DLP), at 68% and 63%, respectively (Table 1). SBP, FBG and HgA1c were higher among the T2DM group (all *p*-values < 0.05), but TC, LDL-C, HDL-C and eGFR were lower (all *p*-values < 0.05; Table 1).

They also used more antihypertensive medications, including calcium channel blockers, angiotensin-converting enzyme inhibitors (ACEIs), angiotensin II receptor blockers (ARBs) and diuretics, along with statins and antiplatelets (all *p*-values < 0.05; Table 2).

### 3.2. Echocardiography Parameters

As tabulated in Table 3, the diabetic group had higher but not statistically significant ARD, LAD, PWT and IVWT values than the control group (*p*-value > 0.05). In terms of the left ventricular (LV) systolic function parameters, LV diameters and EF, the diabetic group had smaller values than the control group (Table 2), but no significant differences were found (*p*-value > 0.05). The patients with T2DM had a significantly higher proportion of diastolic dysfunction, at 48/80 patients (60%), than the controls, at 35/80 subjects (43.7%; *p*-value = 0.04; Table 3).

Multivariate logistic regression of the risk factors associated with diabetes did not show any significant differences in the odds ratio between the two groups (Table 4). However, the odds ratio for HTN was 2.060 with 95% CI (0.862, 4.926), and, for DLP, the odds ratio was 1.643 with 95% CI (0.797, 3.389). Moreover, lower eGFR levels were slightly associated with an increased risk of LVDD in patients with diabetes compared to the control group, but this was not statistically significant.

## 4. Discussion

Diabetic cardiomyopathy (DCM) was coined by Rubler et al. in 1972, who described the condition as myocardial dysfunction in the absence of coronary artery disease, hypertension and alcohol consumption [13]. It varies from subclinical ventricular dysfunction to overt clinical heart failure.

The present study evaluates the effect of DM on systolic function, LV internal dimensions, PWD and the thickness of the interventricular septum (IVS) based on an echocardiographic study. It compares asymptomatic patients with diabetes with a healthy matched control group.

In terms of echocardiographic parameters, ARD, LAD, IVS, PWT, LVIDs and LVIDd values were obtained in this study. Although the IVS, PWT, LVIDd and LVIDs findings were located in the normal global range, ARD and LAD were slightly above the normal range in the diabetic group compared to the non-diabetic group (29 cm vs. 28 cm and 38.3 cm vs. 37.6 cm, respectively). There were no significant differences between the two groups (*p*-value > 0.05).

In the literature, inconsistent results have been established. Our findings are in agreement with those of Zhi et al.’s case–control study, where 48 patients with diabetes were compared with 48 controls, and non-statistically significant measurements were obtained for LVDD, IVSD and PWD: 4.6 ± 0.5 cm vs. 4.5 ± 0.4 cm, 1.1 ± 0.2 cm vs. 1.0 ± 0.2 cm and 1.0 ± 0.1 cm vs. 0.9 ± 0.2 cm, respectively [10]. Another study’s findings agree with our findings, where LVIDs and LVIDd values in 40 patients with diabetes were not statistically significant compared to those of 40 controls [11]. Although Zheo et al.’s case–control study reported no statistically significant differences in ARD between diabetic and healthy populations [14], statistically significant differences were observed in a study among the Sudanese population [15].

EF was another parameter assessed in the current study. Lower values were observed among diabetics than controls, but there were no significant differences between the two groups (*p*-value > 0.05). This is in line with multiple studies in the literature. In the Halil study, EF in the diabetic group did not differ significantly from that in the healthy group [11]. In addition, Mohammed et al. concluded no significant differences in EF between 113 patients with diabetes and 130 controls [15]. Nevertheless, a large cohort study of 2400 patients showed significantly lower EF in the diabetic population than in controls (55 ± 13% vs. 53 ± 13%, respectively), which can be attributed to the larger sample size in addition to the higher age group of their diabetic population (65 ± 10 years) [16].

Our results show significantly higher diastolic dysfunction in the diabetic group than in the control (60% vs. 43.7%, respectively; *p*-value < 0.05). This finding is supported by the majority of studies in the literature. Virendra et al. studied similar patients, with a mean HbA1c of 8.3 ± 1.91% in males and 8.1 ± 1.3% in females, along with a disease duration of 11 ± 5 years in males and 10 ± 4 years in females. The results showed 54.33% diastolic dysfunction among 127 diabetics with normal systolic function compared to 11% among 100 controls (*p*-value < 0.05); patients with a longer disease duration (defined as 11 to 15 years) and an HbA1c of >7.5% were significantly associated with higher diastolic dysfunction (*p*-value < 0.05) [17]. Similarly, significantly higher LV diastolic dysfunction was observed among asymptomatic Indian patients with diabetes than the healthy population [18].

An additional observation was the prevalence of DLP and HTN among our diabetic population at 63% and 68%, respectively. Similar to previous studies, DLP and HTN were highly prevalent in the T2DM patients compared to in the controls: Yaru et al. reported a DLP prevalence of 59.3% in diabetic patients and 39.9% in patients with normal glucose levels [19]; an HTN prevalence of 86.2% was reported among a group of Saudi patients with T2DM by Khalid et al. [20], and an HTN prevalence of 85.6% was reported among Benghazi patients with T2DM by Faiza et al. [21]. The underlying pathophysiology of insulin resistance predisposes patients to metabolic abnormalities, which alter systemic lipid metabolism and result in the development of DLP along with HTN due to inappropriate activation of the renin–angiotensin–aldosterone system and sympathetic nervous system [22,23,24]. The coexistence of DM and HTN may accelerate or worsen several echocardiographic parameters reflecting heart structure or function. Ehud et al. demonstrated a greater LV mass index in hypertensive and diabetic patients than in non-diabetic patients (158 ± 45 g/m^2^ vs. 113 ± 20 g/m^2^) [25]. However, debate is ongoing regarding whether HTN is truly an affecting risk factor. We studied this relation among our population. Our result shows an independent effect of diabetes on diastolic dysfunction despite a significantly higher proportion of DLP and HTN among diabetics vs. non-diabetics. A similar outcome was reported by Micheal et al., who compared the systolic and diastolic functions of 116 diabetic patients with those of 232 matched non-diabetics, as well as coexistent hypertension (51% and 53%, respectively), using echocardiography, and they concluded that DM is an independent risk factor for cardiomyopathy [26].

Several factors could have contributed to the varying results in the literature, i.e., the study design, methods of measuring echocardiographic parameters, age of the sample included, disease duration, coexistence of comorbidities and glycemic control. We relate our findings to the characteristics of our patients. The glycemic control of our diabetic population was moderately controlled (HgA1c: 8.3 ± 1.8%), in addition to the protective effects of statin and antihypertensive medications, including ACEI and ARBs. Poor glycemic control is an observation in multiple studies with positive findings. In case–control studies with significantly higher IVWT, LVDD, PWT and LAD in the diabetics group than in controls, patients were poorly controlled, with a reported mean HgA1c of 11.77 ± 3.53% [14]. A recent metanalysis addressed multiple risk factors associated with DCM, highlighting a cut-off HbA1c of >9% [25]. It was suggested that the regression of diastolic and systolic findings can be achieved with intensive glycemic control. In the study by Melissa et al., blood glucose, blood pressure and cholesterol were optimized to recommended targets for 12 months in subjects with T2DM and poor glycemic control. LV systolic and diastolic functions, measured via LV global longitudinal strain and septal e’ velocities, were shown to improve by 21% and 24%, respectively, with HgA1c improvements of 10.3% ± 2.4% to 8.3% ± 2.0%; patients who achieved an HbA1c of less than 7% showed the greatest improvement [27].

Several well-evidenced metabolic disturbances at the level of the myocardium causing DCM have been suggested, such as hyperglycemia, hyperinsulinemia and insulin resistance resulting in glucotoxicity and lipotoxicity, leading to inflammation, oxidative stress, myocellular hypertrophy, myocardial fibrosis and microvascular dysfunction within the diabetic heart [28,29,30]. This provides a comprehensive picture of subclinical cardiac dysfunction and how it can eventually lead to overt heart failure.

## 5. Study Limitations

### 5.1. Single-Point Observation

Our study was conducted based on retrospective data gathered from medical records containing information on cardiac structural and functional abnormalities at a specific point in the past. This does not offer a dynamic view of how these abnormalities change over time.

### 5.2. Lack of Follow-Up Data

This study did not include any follow-up data, and, as a result, there is no information on how these cardiac abnormalities might have evolved or improved after the initial observation. This limits the ability to draw conclusions about the long-term outcomes of these abnormalities.

### 5.3. Geographical Distribution

As this study was conducted only on patients of King Saud University Medical City, this study’s findings are limited by its population.

### 5.4. Need for Further Studies

To address these limitations and gain a more comprehensive understanding of cardiac abnormalities in T2DM, it is imperative to conduct further studies. These studies can capture the progression, regression or stability of cardiac abnormalities, providing a more dynamic and clinically relevant perspective.

## 6. Conclusions

This is the first retrospective case–control study in Saudi Arabia to assess echocardiographic parameters in T2DM patients compared to those of a healthy population. Diastolic dysfunction among diabetic patients is significantly associated with DM independent of hypertension and dyslipidemia, a finding that supports the concept of DCM. In the asymptomatic period, early screening for DCM should be performed to prevent cardiovascular complications, and preventive measures should be implemented to avoid overt heart failure.

## Figures and Tables

**Table 1:** Comparison of baseline characteristics of cases and controls.

Variables	Cases (n = 80)	Controls (n = 80)	*p*-Value
**Male, n (%)**	35 (43.8%)	35 (43.8%)	1
**Female, n (%)**	45 (56.3%)	45 (56.3%)	
**Duration of diabetes (years)**	13 ± 8.3 years	-	-
**HTN, n (%)**	68 (85%)	47 (58.8%)	0.000
**DLP, n (%)**	63 (78.8%)	25 (31.3%)	0.000
**Age (years)**	58.78 ± 10.23	58.63 ± 10.18	0.926
**Height (cm)**	159.92 ± 9.56	160.13 ± 11.32	0.898
**Weight (kg)**	81.82 ± 15.89	78.03 ± 16.41	0.14
**BMI (kg/m^2^)**	31.68 ± 6.23	30.27 ± 6.61	0.165
**SBP (mmHg)**	133.73 ± 18.82	125.37 ± 19.39	0.006
**DBP (mmHg)**	73.76 ± 11.31	75.01 ± 10.34	0.467
**FBG (mmol/L)**	9.21 ± 3.52	5.99 ± 0.90	0.000
**HbA1c (%)**	8.34 ± 1.83	5.67 ± 0.38	0.000
**TC (mmol/L)**	4.15 ± 1.03	4.60 ± 1.13	0.01
**HDL (mmol/L)**	1.23 ± 0.32	1.39 ± 0.34	0.003
**LDL-C (mmol/L)**	2.27 ± 0.94	2.70 ± 0.89	0.004
**TG (mmol/L)**	1.67 ± 0.97	1.46 ± 0.66	0.105
**Creatinine (μmol/L)**	79.71 ± 24.93	73.15 ± 17.12	0.054
**eGFR (mL/min/1.73 m^2^)**	82.68 ± 19.65	89.35 ± 15.99	0.02

**HTN**: hypertension, **DLP**: dyslipidemia, **BMI**: body mass index, **SBP**: systolic blood pressure, **DBP**: diastolic blood pressure, **FBG**: fasting blood glucose, **HbA1c:** hemoglobin A1C, **TC**: total cholesterol, **HDL**: high-density lipoprotein cholesterol, **LDL-C**: low-density lipoprotein cholesterol, **TG**: triglyceride, **eGFR**: estimated glomerular filtration rate, **n:** number.

**Table 2:** Comparison of antihypertensive medications between cases and controls.

Medications	Cases	Controls	*p*-Value
**BB**	23 (28.7%)	17 (21.2%)	0.103
**CCB**	23 (28.7%)	12 (15%)	0.01
**ARB**	29 (36.2%)	18 (22.5%)	0.02
**ACEI**	15 (18.7%)	7 (8.7%)	0.03
**Diuretic**	28 (35%)	14 (17.5%)	0.006
**Statin**	68 (85%)	28 (35%)	0
**Antiplatelet**	54 (67.5%)	30 (35.7%)	0

**BB**: beta blocker, **CCB**: calcium channel blocker, **ARB**: angiotensin receptor blocker, **ACEI**: angiotensin-converting enzyme inhibitor.

**Table 3:** Comparison of echocardiographic parameters between cases and controls.

Echocardiographic Parameter	Cases (n = 80)	Controls (n = 80)	*p*-Value
**ARD (mm)**	29.03 ± 3.64	28.05 ± 3.39	0.982
**LAD (mm)**	38.30 ± 5.40	37.63 ± 6.87	0.499
**LVIDd (mm)**	46.46 ± 5.61	47.11 ± 6.15	0.486
**LVIDs (mm)**	27.73 ± 6.99	28.46 ± 5.53	0.469
**PWT (mm)**	9.32 ± 9.9	8.33 ± 1.57	0.381
**IVWT (mm)**	9.92 ± 2.23	9.38 ± 2.66	0.169
**EF (%)**	55.50 ± 8.84	56.25 ± 8.28	0.581
**LVDD, n (%)**	48 (60%)	35 (43.7%)	0.04

**ARD**: aortic root diameter, **LAD**: left atrium diameter, **LVIDd**: left ventricular internal diameter end diastole, **LVIDs**: left ventricular internal diameter end systole, **PWT**: posterior wall thickness, **IVWT**: interventricular wall thickness, **EF**: ejection fraction, **LVDD:** left ventricular diastolic dysfunction, **n:** number.

**Table 4:** Multivariate logistic regression of risk factors associated with DM.

Factor	B	Exp(B) OR	Sig.	95% C.I.
HTN	0.723	**2.060**	0.104	0.862–4.926
DLP	0.497	**1.643**	0.179	0.797–3.389
SBP	0.013	**1.013**	0.190	0.994–1.033
TC	0.457	**1.580**	0.184	0.805–3.102
HDL-C	−0.015	**0.985**	0.977	0.352–2.756
LDL-C	−0.589	**0.555**	0.143	0.252–1.220
eGFR	−0.021	**0.979**	0.058	0.958–1.001

**HTN**: hypertension, **DLP**: dyslipidemia, **SBP**: systolic blood pressure, **TC**: total cholesterol, **HDL**: high-density lipoprotein cholesterol, **LDL-C**: low-density lipoprotein cholesterol, **eGFR**: estimated glomerular filtration rate.

## Data Availability

The data used to support the findings of this study are restricted by the institutional review board at King Saud University Medical City to protect patient privacy.
